# Supplementary figures and images for: Effect of Soil Type: Qualitative and Quantitative Analysis of Phytochemicals in Some Browse Species Leaves Found in Savannah Biome of South Africa
Source: Molecules. 2022 Feb 22;27(5):1462. doi: 10.3390/molecules27051462 (PMC8911906; doi:10.3390/molecules27051462)

Supplementary material: Calibration for tannins

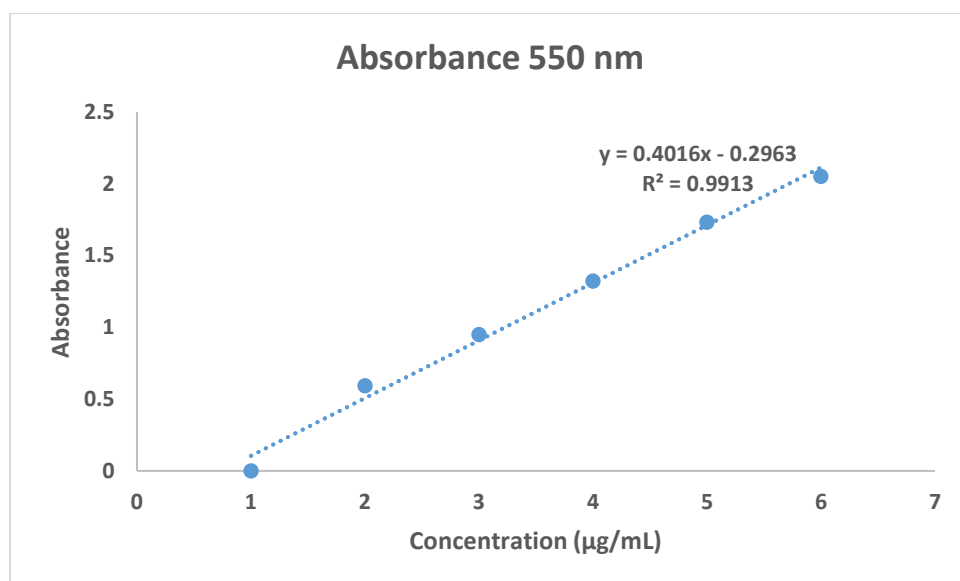

Supplement: Supplementary file 1 [file molecules-27-01462-s001.zip › molecules-1586808-supplementary.pdf]
